# 3,4-Di­methyl­phenyl benzoate

**DOI:** 10.1107/S1600536814001299

**Published:** 2014-01-22

**Authors:** Rodolfo Moreno-Fuquen, Mauricio Rendón, Alan R. Kennedy

**Affiliations:** a Departamento de Química - Facultad de Ciencias, Universidad del Valle, Apartado 25360, Santiago de Cali, Colombia; b WestCHEM, Department of Pure and Applied Chemistry, University of Strathclyde, 295 Cathedral Street, Glasgow G1 1XL, Scotland

## Abstract

In the title compound, C_15_H_14_O_2_, the terminal rings form a dihedral angle of 52.39 (4)°. The mean plane of the central ester group [r.m.s. deviation = 0.0488 Å] is twisted away from the benzene and phenyl rings by 60.10 (4) and 8.67 (9)°, respectively. In the crystal, mol­ecules are linked by weak C—H⋯O hydrogen bonds, forming *C*(6) chains which run along [100].

## Related literature   

For similar structures, see: Gowda *et al.* (2008*a* ▶,*b* ▶). For hydrogen-bonding information, see: Nardelli (1995 ▶) and for hydrogen-bond motifs, see: Etter *et al.* (1990 ▶).
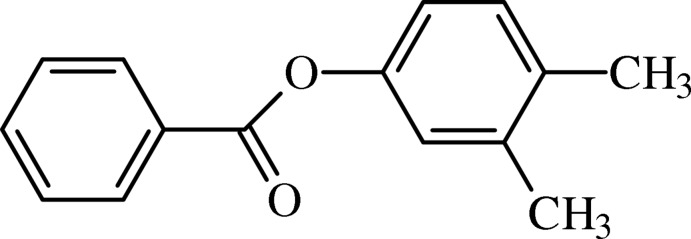


## Experimental   

### 

#### Crystal data  

C_15_H_14_O_2_
*M* *_r_* = 226.26
Triclinic, 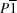

*a* = 6.0293 (4) Å
*b* = 7.8506 (3) Å
*c* = 13.1163 (9) Å
α = 88.592 (4)°
β = 77.020 (5)°
γ = 77.680 (4)°
*V* = 590.87 (6) Å^3^
*Z* = 2
Mo *K*α radiation
μ = 0.08 mm^−1^
*T* = 123 K
0.33 × 0.25 × 0.06 mm

#### Data collection  

Oxford Diffraction Xcalibur E diffractometer
Absorption correction: multi-scan (*CrysAlis PRO*; Oxford Diffraction, 2010 ▶) *T*_min_ = 0.977, *T*_max_ = 1.000
5782 measured reflections
2965 independent reflections
2196 reflections with *I* > 2σ(*I*)
*R*_int_ = 0.021

#### Refinement  

*R*[*F* ^2^ > 2σ(*F* ^2^)] = 0.048
*wR*(*F* ^2^) = 0.112
*S* = 1.04
2965 reflections
156 parameters
H-atom parameters constrained
Δρ_max_ = 0.28 e Å^−3^
Δρ_min_ = −0.24 e Å^−3^

### 

Data collection: *CrysAlis PRO* (Oxford Diffraction, 2010 ▶); cell refinement: *CrysAlis PRO*; data reduction: *CrysAlis PRO*; program(s) used to solve structure: *SHELXS97* (Sheldrick, 2008 ▶); program(s) used to refine structure: *SHELX97* (Sheldrick, 2008 ▶); molecular graphics: *ORTEP-3 for Windows* (Farrugia, 2012 ▶) and *Mercury* (Macrae *et al.*, 2006 ▶); software used to prepare material for publication: *WinGX* (Farrugia, 2012 ▶).

## Supplementary Material

Crystal structure: contains datablock(s) I, global. DOI: 10.1107/S1600536814001299/tk5288sup1.cif

Structure factors: contains datablock(s) I. DOI: 10.1107/S1600536814001299/tk5288Isup2.hkl

Supporting information file. DOI: 10.1107/S1600536814001299/tk5288Isup3.cml

CCDC reference:

Additional supporting information:  crystallographic information; 3D view; checkCIF report

## Figures and Tables

**Table 1:** Hydrogen-bond geometry (Å, °)

*D*—H⋯*A*	*D*—H	H⋯*A*	*D*⋯*A*	*D*—H⋯*A*
C2—H2⋯O8^i^	0.95	2.47	3.3710 (18)	157

Symmetry code: (i) 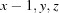.

**Fig. 1.**
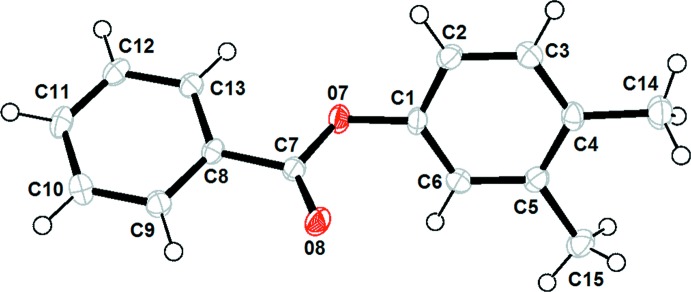
Molecular conformation and atom numbering scheme for the title compound with displacement ellipsoids drawn at the 50% probability level. H atoms are shown as spheres of arbitrary radius.

**Fig. 2.**
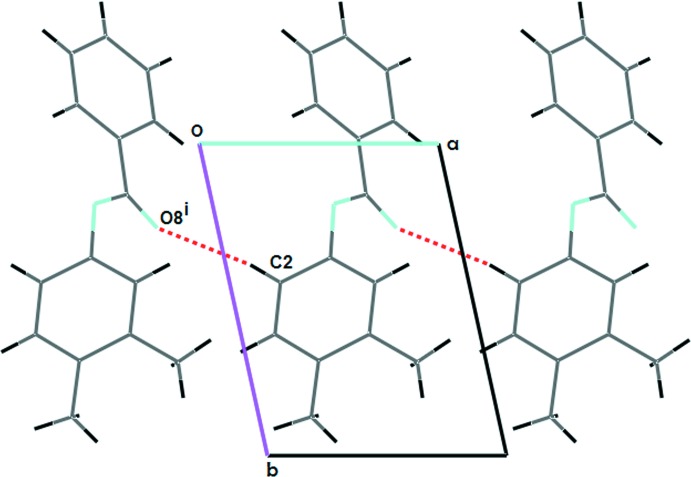
Part of the crystal structure of (I), showing the formation of chains which running along [100]. Symmetry code: (i) x-1, +y, +z.

## supplementary crystallographic information

### 2.1. Synthesis and crystallization

The reagents and solvents for the synthesis were obtained from Aldrich Chemical Co., and were used without additional purification. The title molecule was synthesized using equimolar qu­anti­ties of 3,4-di­methyl­phenol and benzoyl chloride. 3,4-Di­methyl­phenol (0.50 g, 4.10 mmol) was added to a solution of anhydrous aluminum chloride (0.40 g, 3.00 mmol) in anhydrous di­chloro­methane (25 mL). The resulting solution was cooled and benzoyl chloride (0.57 g) was added slowly at 0-5°. After complete addition, the mixture was left under stirring at room temperature for 0.5 h, and then it was heated (reflux) to 50° C for 1 h. The reaction mixture was poured onto ice (100 g). The crude product was isolated by extraction with di­chloro­methane, and it was separated. The solution was dried over Na_2_SO_4_ and it was evaporated at room temperature. The obtained amorphous product was dissolved in methanol and the solution was left to slow evaporation. Colourless crystals of good quality were obtained with M.pt = 322 (1) K.

### 2.2. Refinement

All H-atoms were positioned at geometrically idealized positions with C—H distance of 0.95–0.98 Å, and with *U*_iso_(H) = 1.2–1.5*U*_eq_ of the C-atoms to which they were bonded.

### 3. Results and discussion

In order to obtain more detailed information about the effect of substitution of the methyl groups on the structure of benzoate system, the structure determination of 3,4-di­methyl phenyl benzoate (I) has been carried out. Two very similar molecular structures, 2,3-di­methyl­phenyl benzoate, DMPB1 (Gowda *et al.*, 2008*a*), and 2,4-di­methyl­phenyl benzoate, DMPB2 (Gowda *et al.*, 2008*b*), were taken for comparison with the structure (I). The rings of (I), Fig. 1, form a dihedral angle of 52.39 (4)° while in DMPB1 and DMPB2 they form dihedral angles of 87.36 (6)° and 80.25 (5)°, respectively. The other bond lengths and bond angles of DMPB1 and DMPB2 are close to the title system. The central ester moiety (C1—O7—C7(O8)—C8) is twisted away from the di­methyl-substituted benzene­and phenyl rings by 60.10 (4) and 8.67 (9)°, respectively. The crystal packing shows no classical hydrogen bonds and it is stabilized by weak C—H···O inter­molecular hydrogen bonds, forming *C*(6) chains (Etter, 1990) along [100] (see Fig. 2). The C2 atom acts as hydrogen-bond donor to O1 atom at (*x*-1, +*y*, +*z*) (Nardelli, 1995); see Table 1.

### Crystal data

**Table:**

C_15_H_14_O_2_	*Z* = 2
*M**_r_* = 226.26	*F*(000) = 240
Triclinic, *P*1	*D*_x_ = 1.272 Mg m^−^^3^
Hall symbol: -P 1	Melting point: 322(1) K
*a* = 6.0293 (4) Å	Mo *K*α radiation, λ = 0.71073 Å
*b* = 7.8506 (3) Å	Cell parameters from 2013 reflections
*c* = 13.1163 (9) Å	θ = 3.1–29.6°
α = 88.592 (4)°	µ = 0.08 mm^−^^1^
β = 77.020 (5)°	*T* = 123 K
γ = 77.680 (4)°	Plate, colourless
*V* = 590.87 (6) Å^3^	0.33 × 0.25 × 0.06 mm

### Data collection

**Table:**

Oxford Diffraction Xcalibur E diffractometer	2965 independent reflections
Radiation source: fine-focus sealed tube	2196 reflections with *I* > 2σ(*I*)
Graphite monochromator	*R*_int_ = 0.021
ω scans	θ_max_ = 29.8°, θ_min_ = 3.1°
Absorption correction: multi-scan (*CrysAlis PRO*; Oxford Diffraction, 2010)	*h* = −8→8
*T*_min_ = 0.977, *T*_max_ = 1.000	*k* = −10→10
5782 measured reflections	*l* = −18→18

### Refinement

**Table:**

Refinement on *F*^2^	Primary atom site location: structure-invariant direct methods
Least-squares matrix: full	Secondary atom site location: difference Fourier map
*R*[*F*^2^ > 2σ(*F*^2^)] = 0.048	Hydrogen site location: inferred from neighbouring sites
*wR*(*F*^2^) = 0.112	H-atom parameters constrained
*S* = 1.04	*w* = 1/[σ^2^(*F*_o_^2^) + (0.0425*P*)^2^ + 0.1253*P*] where *P* = (*F*_o_^2^ + 2*F*_c_^2^)/3
2965 reflections	(Δ/σ)_max_ < 0.001
156 parameters	Δρ_max_ = 0.28 e Å^−^^3^
0 restraints	Δρ_min_ = −0.24 e Å^−^^3^

### Special details

**Table:**

Geometry. All esds (except the esd in the dihedral angle between two l.s. planes) are estimated using the full covariance matrix. The cell esds are taken into account individually in the estimation of esds in distances, angles and torsion angles; correlations between esds in cell parameters are only used when they are defined by crystal symmetry. An approximate (isotropic) treatment of cell esds is used for estimating esds involving l.s. planes.
Refinement. Refinement of *F*^2^ against ALL reflections. The weighted *R*-factor *wR* and goodness of fit *S* are based on *F*^2^, conventional *R*-factors *R* are based on *F*, with *F* set to zero for negative *F*^2^. The threshold expression of *F*^2^ > σ(*F*^2^) is used only for calculating *R*-factors(gt) *etc*. and is not relevant to the choice of reflections for refinement. *R*-factors based on *F*^2^ are statistically about twice as large as those based on *F*, and *R*- factors based on ALL data will be even larger.

### Fractional atomic coordinates and isotropic or equivalent isotropic displacement parameters (Å^2^)

**Table:**

	*x*	*y*	*z*	*U*_iso_*/*U*_eq_	
O7	0.50750 (18)	0.19314 (11)	0.74671 (8)	0.0239 (2)	
O8	0.74671 (18)	0.27031 (12)	0.83880 (8)	0.0262 (3)	
C1	0.4447 (2)	0.36566 (16)	0.71295 (11)	0.0202 (3)	
C2	0.2126 (2)	0.44540 (17)	0.74124 (11)	0.0227 (3)	
H2	0.1032	0.3888	0.7848	0.027*	
C3	0.1425 (3)	0.61121 (18)	0.70426 (11)	0.0236 (3)	
H3	−0.0171	0.6682	0.7231	0.028*	
C4	0.3009 (3)	0.69544 (17)	0.64030 (11)	0.0218 (3)	
C5	0.5366 (2)	0.61144 (17)	0.61281 (11)	0.0213 (3)	
C6	0.6075 (2)	0.44503 (17)	0.64949 (11)	0.0211 (3)	
H6	0.7665	0.3865	0.6310	0.025*	
C7	0.6508 (2)	0.16320 (16)	0.81492 (10)	0.0184 (3)	
C8	0.6695 (2)	−0.01620 (16)	0.85620 (10)	0.0182 (3)	
C9	0.8327 (2)	−0.07177 (17)	0.91616 (11)	0.0226 (3)	
H9	0.9322	0.0019	0.9267	0.027*	
C10	0.8508 (3)	−0.23504 (18)	0.96080 (12)	0.0257 (3)	
H10	0.9613	−0.2727	1.0025	0.031*	
C11	0.7073 (3)	−0.34264 (17)	0.94426 (11)	0.0252 (3)	
H11	0.7192	−0.4542	0.9748	0.030*	
C12	0.5461 (3)	−0.28836 (17)	0.88330 (11)	0.0241 (3)	
H12	0.4495	−0.3635	0.8716	0.029*	
C13	0.5251 (2)	−0.12484 (16)	0.83937 (11)	0.0205 (3)	
H13	0.4135	−0.0872	0.7982	0.025*	
C14	0.2186 (3)	0.87556 (18)	0.60106 (13)	0.0314 (4)	
H14A	0.0507	0.9147	0.6291	0.047*	
H14B	0.2509	0.8716	0.5244	0.047*	
H14C	0.3010	0.9570	0.6242	0.047*	
C15	0.7122 (3)	0.6999 (2)	0.54453 (12)	0.0292 (3)	
H15A	0.8692	0.6291	0.5400	0.044*	
H15B	0.7010	0.8151	0.5748	0.044*	
H15C	0.6807	0.7130	0.4743	0.044*	

### Atomic displacement parameters (Å^2^)

**Table:**

	*U*^11^	*U*^22^	*U*^33^	*U*^12^	*U*^13^	*U*^23^
O7	0.0319 (6)	0.0159 (4)	0.0298 (6)	−0.0087 (4)	−0.0163 (5)	0.0063 (4)
O8	0.0332 (6)	0.0197 (5)	0.0327 (6)	−0.0133 (4)	−0.0150 (5)	0.0059 (4)
C1	0.0273 (8)	0.0157 (6)	0.0211 (7)	−0.0066 (5)	−0.0112 (6)	0.0045 (5)
C2	0.0236 (7)	0.0247 (7)	0.0220 (7)	−0.0100 (6)	−0.0057 (6)	0.0049 (6)
C3	0.0222 (7)	0.0248 (7)	0.0240 (7)	−0.0035 (6)	−0.0071 (6)	0.0014 (6)
C4	0.0283 (8)	0.0189 (6)	0.0206 (7)	−0.0050 (6)	−0.0111 (6)	0.0028 (5)
C5	0.0256 (8)	0.0232 (7)	0.0181 (7)	−0.0097 (6)	−0.0074 (6)	0.0033 (5)
C6	0.0204 (7)	0.0226 (7)	0.0207 (7)	−0.0041 (5)	−0.0058 (6)	0.0001 (5)
C7	0.0194 (7)	0.0174 (6)	0.0188 (7)	−0.0051 (5)	−0.0043 (5)	0.0016 (5)
C8	0.0216 (7)	0.0147 (6)	0.0175 (7)	−0.0042 (5)	−0.0025 (5)	−0.0001 (5)
C9	0.0261 (8)	0.0196 (6)	0.0249 (7)	−0.0067 (5)	−0.0097 (6)	0.0012 (6)
C10	0.0303 (8)	0.0225 (7)	0.0241 (7)	−0.0013 (6)	−0.0098 (6)	0.0030 (6)
C11	0.0322 (8)	0.0147 (6)	0.0251 (8)	−0.0034 (6)	−0.0012 (6)	0.0034 (5)
C12	0.0278 (8)	0.0183 (6)	0.0264 (8)	−0.0094 (6)	−0.0024 (6)	−0.0003 (6)
C13	0.0217 (7)	0.0182 (6)	0.0225 (7)	−0.0053 (5)	−0.0056 (6)	0.0002 (5)
C14	0.0376 (9)	0.0237 (7)	0.0361 (9)	−0.0062 (6)	−0.0161 (7)	0.0080 (6)
C15	0.0323 (9)	0.0324 (8)	0.0270 (8)	−0.0145 (7)	−0.0087 (7)	0.0101 (6)

### Geometric parameters (Å, º)

**Table:**

O7—C7	1.3609 (16)	C8—C13	1.3950 (18)
O7—C1	1.4145 (15)	C9—C10	1.3892 (19)
O8—C7	1.2008 (16)	C9—H9	0.9500
C1—C2	1.3755 (19)	C10—C11	1.384 (2)
C1—C6	1.383 (2)	C10—H10	0.9500
C2—C3	1.3909 (19)	C11—C12	1.386 (2)
C2—H2	0.9500	C11—H11	0.9500
C3—C4	1.391 (2)	C12—C13	1.3867 (18)
C3—H3	0.9500	C12—H12	0.9500
C4—C5	1.402 (2)	C13—H13	0.9500
C4—C14	1.5133 (18)	C14—H14A	0.9800
C5—C6	1.3943 (19)	C14—H14B	0.9800
C5—C15	1.504 (2)	C14—H14C	0.9800
C6—H6	0.9500	C15—H15A	0.9800
C7—C8	1.4871 (17)	C15—H15B	0.9800
C8—C9	1.3872 (19)	C15—H15C	0.9800
			
C7—O7—C1	117.97 (10)	C8—C9—H9	120.0
C2—C1—C6	122.09 (13)	C10—C9—H9	120.0
C2—C1—O7	116.62 (12)	C11—C10—C9	119.75 (14)
C6—C1—O7	121.17 (12)	C11—C10—H10	120.1
C1—C2—C3	118.16 (13)	C9—C10—H10	120.1
C1—C2—H2	120.9	C10—C11—C12	120.31 (13)
C3—C2—H2	120.9	C10—C11—H11	119.8
C2—C3—C4	121.48 (14)	C12—C11—H11	119.8
C2—C3—H3	119.3	C11—C12—C13	120.32 (13)
C4—C3—H3	119.3	C11—C12—H12	119.8
C3—C4—C5	119.23 (12)	C13—C12—H12	119.8
C3—C4—C14	120.13 (13)	C12—C13—C8	119.38 (13)
C5—C4—C14	120.64 (13)	C12—C13—H13	120.3
C6—C5—C4	119.46 (13)	C8—C13—H13	120.3
C6—C5—C15	120.11 (13)	C4—C14—H14A	109.5
C4—C5—C15	120.43 (13)	C4—C14—H14B	109.5
C1—C6—C5	119.57 (13)	H14A—C14—H14B	109.5
C1—C6—H6	120.2	C4—C14—H14C	109.5
C5—C6—H6	120.2	H14A—C14—H14C	109.5
O8—C7—O7	123.50 (12)	H14B—C14—H14C	109.5
O8—C7—C8	125.00 (13)	C5—C15—H15A	109.5
O7—C7—C8	111.50 (11)	C5—C15—H15B	109.5
C9—C8—C13	120.17 (12)	H15A—C15—H15B	109.5
C9—C8—C7	117.48 (12)	C5—C15—H15C	109.5
C13—C8—C7	122.32 (12)	H15A—C15—H15C	109.5
C8—C9—C10	120.06 (13)	H15B—C15—H15C	109.5
			
C7—O7—C1—C2	−115.77 (14)	C1—O7—C7—O8	−8.5 (2)
C7—O7—C1—C6	67.97 (17)	C1—O7—C7—C8	170.72 (11)
C6—C1—C2—C3	−0.1 (2)	O8—C7—C8—C9	−8.7 (2)
O7—C1—C2—C3	−176.31 (12)	O7—C7—C8—C9	172.01 (12)
C1—C2—C3—C4	0.1 (2)	O8—C7—C8—C13	169.10 (14)
C2—C3—C4—C5	−0.2 (2)	O7—C7—C8—C13	−10.15 (18)
C2—C3—C4—C14	179.75 (13)	C13—C8—C9—C10	−0.8 (2)
C3—C4—C5—C6	0.4 (2)	C7—C8—C9—C10	177.06 (13)
C14—C4—C5—C6	−179.59 (13)	C8—C9—C10—C11	0.7 (2)
C3—C4—C5—C15	−179.47 (13)	C9—C10—C11—C12	0.1 (2)
C14—C4—C5—C15	0.6 (2)	C10—C11—C12—C13	−0.8 (2)
C2—C1—C6—C5	0.3 (2)	C11—C12—C13—C8	0.7 (2)
O7—C1—C6—C5	176.31 (12)	C9—C8—C13—C12	0.1 (2)
C4—C5—C6—C1	−0.4 (2)	C7—C8—C13—C12	−177.65 (12)
C15—C5—C6—C1	179.46 (13)		

### Hydrogen-bond geometry (Å, º)

**Table:**

*D*—H···*A*	*D*—H	H···*A*	*D*···*A*	*D*—H···*A*
C2—H2···O8^i^	0.95	2.47	3.3710 (18)	157

Symmetry code: (i) *x*−1, *y*, *z*.
